# Dietary behavior patterns and short-term pace of aging—evidence from a Chinese cohort

**DOI:** 10.3389/fnut.2026.1924515

**Published:** 2026-09-09

**Authors:** Xin Huang, Zhaoyang Zhou, Pingting Yang, Jianzhou Yang, Yongmei He, Lu Yin, Jing Deng, Ying Li

**Affiliations:** 1 Department of Epidemiology, School of Public Health, Health Science Center, Hunan Normal University, Changsha, Hunan, China; 2 Department of Health Management, The Third Xiangya Hospital, Central South University, Changsha, China; 3 Department of Epidemiology and Biostatistics, School of Public Health, Xi’an Jiaotong University Health Science Center, Xi’an, Shaanxi, China; 4 School of Public Health, Changzhi Medical College, Changzhi, China; 5 Department of Health Management, Aerospace Center Hospital, Beijing, China; 6 Information Center, Fuwai Hospital, Chinese Academy of Medical Sciences & Peking Union Medical College, National Center for Cardiovascular Diseases, Beijing, China; 7 Department of Epidemiology, Xiangya School of Public Health, Central South University, Changsha, Hunan, China

**Keywords:** aging pace, biological age, dietary behavior, latent class analysis, prospective cohort

## Abstract

**Background:**

Unhealthy dietary behaviors have been associated with increased risks of mortality and chronic diseases, yet mortality is a distal endpoint that requires long-term follow-up and is therefore poorly suited for short-term evaluation. Aging, which lies upstream of mortality and is modifiable by lifestyle factors, may serve as a meaningful early intermediate outcome. However, evidence linking dietary behavior patterns to short-term changes in the pace of biological aging remains limited. Therefore, we conducted this study to examine the associations between dietary behavior patterns and short-term changes in the pace of biological aging in a Chinese adult population, and to assess whether these associations vary by follow-up duration and chronological age.

**Methods:**

In a longitudinal cohort from two health management centers in China, dietary behavior patterns were identified using data-driven clustering approaches and categorized into low-, moderate-, and high-risk groups. Changes in biological age, estimated from multi-system biomarkers, were used to quantify short-term aging pace. Associations over 12- and 24-month follow-up periods were assessed using weighted longitudinal models.

**Results:**

Among 71,579 participants in the longitudinal cohort, higher-risk dietary behavior patterns were associated with greater increases in biological age over 12 months. When dietary behavior pattern was modeled as an ordinal exposure, each per-level increase in dietary risk was associated with a 0.017-year higher change in biological age at 12 months (95% CI: 0.003, 0.030). In contrast, no statistically significant associations were observed at 24 months. Associations were generally consistent across chronological age groups. Exploratory analyses of individual behavioral dimensions showed similar short-term patterns.

**Conclusion:**

Adverse dietary behavior patterns were associated with modest but detectable short-term acceleration in biological aging, with attenuation over time. These findings support the use of short-term biological aging measures as intermediate outcomes for evaluating early physiological responses to dietary behaviors.

## Introduction

Previous studies from our team have demonstrated that unhealthy dietary behaviors, including irregular eating patterns, preference for salty or high-fat foods, and unbalanced dietary structure are strongly associated with increased risks of all-cause and cardiovascular mortality (1). Although mortality in general population is a meaningful endpoint from a public health perspective, it typically requires long-term follow-up to be observed, limiting its utility for the short-term evaluation of dietary behavior interventions. This highlights the need for a sensitive and potentially reversible intermediate indicator that can reflect changes in overall health status.

Aging is the strongest risk factor for death and most chronic diseases and represents a fundamental biological process underlying long-term health outcomes. Importantly, the aging process is not fixed but can be shaped by environmental and behavioral factors (2), including physical activity, nutrition, and other lifestyle characteristics (3–6). As a modifiable process that lies upstream of mortality, aging provides a plausible intermediate framework linking dietary behaviors to long-term health outcomes (7).

Most prior research on diet and aging has focused on food quantity (8), specific nutrients (9, 10), or overall dietary patterns defined by nutrient composition or diet type (11–14). In recent years, several studies have begun to examine individual dietary habits (15–17), such as preference for salty foods, regular consumption of sugar-sweetened beverages, and frequent fast-food consumption, and have reported associations with accelerated aging or aging-related outcomes. However, such behaviors rarely occur in isolation (18, 19). Eating-related behaviors, including meal timing irregularity, late-night eating, food preferences, and discretionary eating practices such as frequent eating out, tend to co-occur and cluster within individuals, forming distinct behavioral profiles that may capture dimensions of dietary exposure not reflected by conventional nutrient- or food-based measures. Evidence linking these behavior-based patterns to aging and mortality, nevertheless, remains limited.

In China, rapid socioeconomic development over recent decades has been accompanied by substantial shifts in dietary behaviors (20, 21), including increased consumption of sugar-sweetened beverages among younger adults, widespread use of food delivery services, and more frequent late-night eating. These contemporary dietary behavior patterns differ markedly from those typically observed in Western populations, underscoring the need for population-specific evidence. Moreover, it remains unclear whether the influence of dietary behavior patterns on the aging process is persistent over time, accumulates with prolonged exposure, or attenuates as individuals adapt, particularly over short follow-up periods.

Against this background, we conducted a cohort study in a Chinese population to derive dietary behavior patterns, evaluate their associations with short-term changes in the pace of aging, and examine potential time trends and age-related effect modification.

## Methods

### Study population

We used routinely collected health check-up data from two health management centers in China, the Aerospace Center Hospital in Beijing and the Third Xiangya Hospital, Central South University, in Changsha. A total of 341,530 examinations were conducted in Changsha between Jan 2017 and Dec 2023 and 66,038 examinations were conducted in Beijing between Jan 2020 and Dec 2022, together constituting the source population for the present study.

To ensure that the population used for the derivation of the biological age measure and dietary behavior patterns did not overlap with the longitudinal cohort used for association analyses, participants were first classified according to the availability of repeated examinations.

Individuals who had only a single eligible examination during the study period were considered for inclusion in a cross-sectional sample, which was used for the construction of the aging measures and the derivation of dietary behavior patterns. And individuals with at least two eligible examinations were considered for inclusion in the longitudinal cohort. For these participants, the earliest eligible examination was designated as baseline. Follow-up examinations were defined using two pre-specified windows at 10–14 months (12-month window) and 22–26 months (24-month window) after baseline. These windows were selected to capture short-term changes in the pace of aging while allowing evaluation of potential temporal persistence or attenuation of associations over a modest extension of follow-up. Eligible participants were aged ≥18 years at baseline, had available dietary behavior data and biomarkers required for aging assessment, and had no history of pregnancy, fatal cardiovascular disease, cancer diagnosis, or lipid-lowering medication use at baseline.

**Figure 1:**
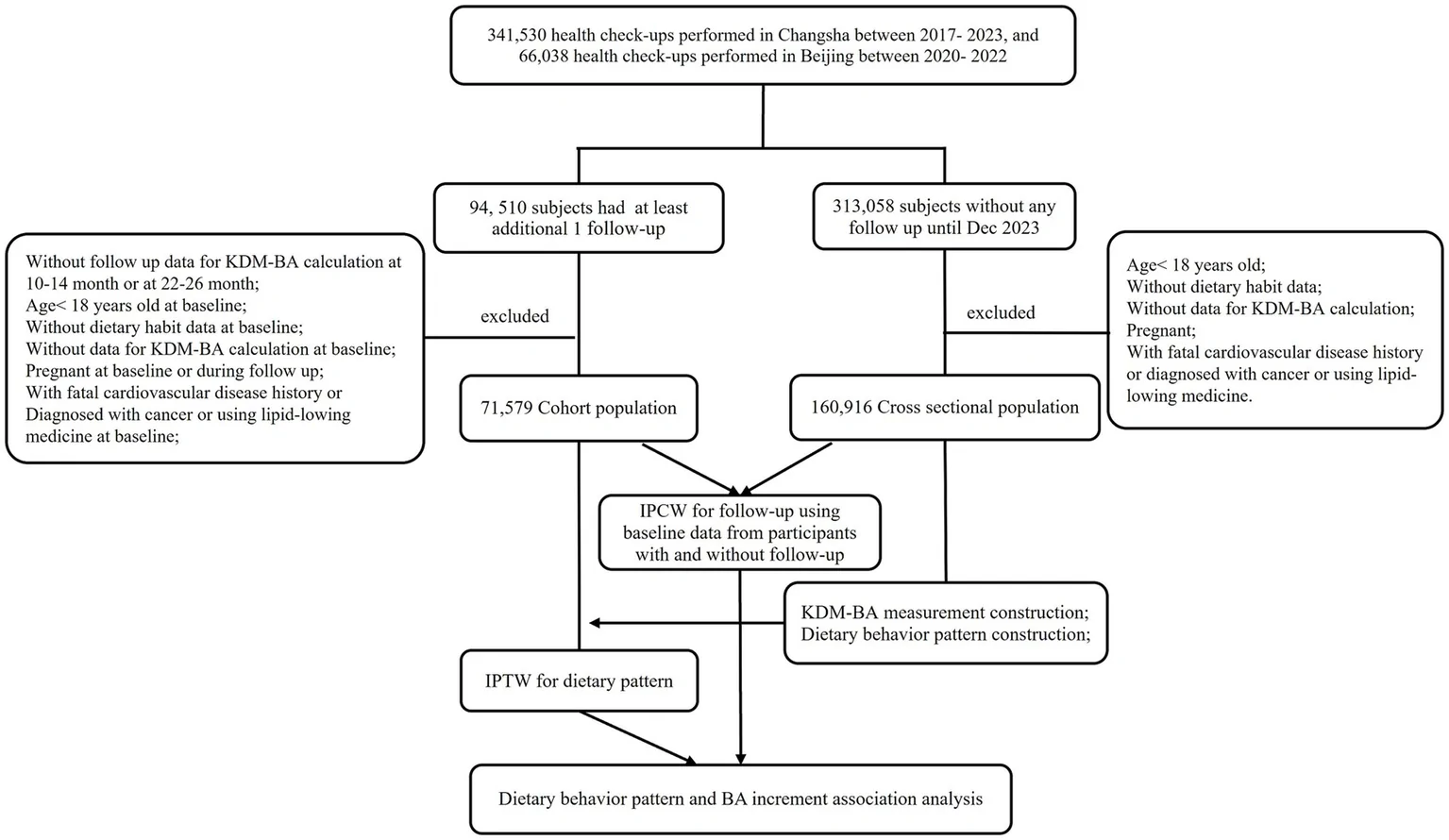
Study flowchart. BA, biological age; KDM, Klemera and Doubal method; IPTW, inverse probability of treatment weighting; IPCW, inverse probability of censoring weighting.

Details of eligibility assessment and exclusion at each stage are shown in Figure 1. The final analytic samples included 160,916 individuals aged 18–82 years in the cross-sectional dataset and 71,579 individuals aged 18–81 years in the longitudinal cohort. All data were routinely collected, fully de-identified prior to analysis, and analyzed in accordance with relevant ethical guidelines. The study protocol was approved by the Institutional Review Board of the Third Xiangya Hospital and Aerospace Center Hospital, with a waiver of informed consent.

### Data collection

Routine health check-ups included a self-administered questionnaire, standardized physical examinations, and fasting venous blood tests. The questionnaire collected information on chronological age (CA), sex, smoking and alcohol consumption, dietary behaviors, physical activity, medication use, and prior medical diagnoses. Questionnaire responses were verified by physicians during the examination.

Blood samples were analyzed for hematologic, metabolic, hepatic, renal, lipid, glycemic, and cardiovascular-related biomarkers. When glycated hemoglobin (HbA1c) was unavailable, it was estimated from fasting blood glucose using a validated linear transformation: HbA1C = (glucose+46.7)/28.7 (22). For participants with multiple examinations, baseline values were defined using the earliest eligible visit with complete dietary and biomarker data.

### Measurement of aging using a biomarker-based biological age index

To quantify the aging process, we operationalized aging using a biomarker-based biological age index derived from the Klemera–Doubal method (KDM), following previously published algorithms and reference code^1^ (23). The KDM approach integrates information from multiple physiological systems to estimate an individual’s biological aging status.

Using the cross-sectional dataset, a validated KDM-based biological age model was derived based on multisystem biomarkers and demonstrated strong correlation with chronological age, low prediction error, and good calibration. Details of biomarker selection, model construction, performance and validation procedures are provided in the Supplementary methods S1 and Supplementary Tables S1–S4.

In the longitudinal cohort, missing biomarker values were imputed using age- and sex-specific means at baseline and last observation carried forward for follow-up visits. Biological age was calculated at baseline and follow-up examinations using the derived model.

### Dietary behavior pattern derivation

Dietary behaviors were assessed using an online self-reported questionnaire with 18 questions (Supplementary Table S5), from which 23 dietary behavior variables consistently available across the study period were derived, covering eating regularity, food preferences, and discretionary eating practices. Using the cross-sectional dataset, principal component analysis (PCA) with oblique rotation was first applied to summarize correlated behaviors into latent behavioral dimensions (Supplementary Figure S1). PCA identified three dietary factors: discretionary/irregular eating & salty–eating-out, the low-quality diet and the high-fat animal foods preference (Supplementary Tables S6, S7).

Latent class analysis (LCA) was subsequently performed based on PCA-derived factor scores (Supplementary methods S2). Among the candidate models, the three-class solution was selected based on model fit, classification quality, and interpretability, with the lowest BIC (329.66), adjusted BIC (266.10), and acceptable entropy (0.76). The average posterior probabilities for the three classes were 0.999, 0.942, and 0.766, respectively (Supplementary Table S8). Based on the class-specific response probabilities, the low-risk pattern was characterized by predominantly low levels across all three dietary dimensions, the moderate-risk pattern by moderate discretionary/irregular and salty/eating-out behaviors together with moderately elevated low-quality and high-fat animal-food preferences, and the high-risk pattern by high levels across all three dimensions (Supplementary Table S9). The derived LCA structure was transported to the longitudinal cohort using posterior class probabilities, and participants were assigned to the most likely dietary behavior pattern group.

### Statistical analyses

Baseline characteristics were compared across dietary behavior pattern groups using standardized mean differences (SMDs). To estimate the short-term association between baseline dietary behavior patterns and subsequent changes in the pace of aging, analyses were conducted under a weighted pseudo-population in which baseline characteristics were balanced and selective follow-up was accounted for.

Stabilized inverse probability of treatment weighting (IPTW) was applied using a multinomial logistic regression model including demographic, behavioral, and clinical covariates (Supplementary methods S3). Because study center represented the recruitment setting rather than an individual-level baseline characteristic, it was not included in the IPTW model. However, considering potential regional differences in dietary behaviors, study center was additionally adjusted for in all outcome models. To account for selective follow-up, stabilized inverse probability of censoring weights (IPCW) were estimated in the baseline-eligible population regardless of follow-up completion. Final analysis weights were defined as the product of stabilized IPTW and IPCW and truncated at the 1st and 99th percentiles.

The primary outcome was short-term change in the pace of aging, operationalized as the change in biological age (Δ*KDM-BA*) between baseline and follow-up. Weighted generalized estimating equations (GEE) were used to estimate associations between dietary behavior patterns and Δ*KDM-BA* across the two follow-up windows, with additional adjustment for baseline chronological age and study center. Interaction terms were included to explore potential effect modification by follow-up time and chronological age at baseline. Secondary analyses examined associations between individual PCA-derived behavioral dimensions and changes in the pace of aging. Sensitivity analyses excluded participants with baseline hypertension or diabetes. KDM-based biological age derivation was conducted using R 4.4.3, and weighting and regression analyses were conducted using SAS 9.4. All analyses were two-sided with a significance level of 𝛼=0.05.

## Results

Using the LCA solution derived from the cross-sectional dataset, 33.89, 33.11, and 33.00% of the cohort participants were classified into the low-, moderate-, and high-risk dietary patterns, respectively. Baseline characteristics differed substantially across the three groups. Individuals with higher-risk dietary patterns were more likely to be younger, have lower KDM-BA, be male, unmarried, smokers, drinkers, with inadequate physical activities, and without a history of DM and hypertension (*P*_*for* trend_ <0.05). After applying IPTW, covariates balance improved markedly, with all weighted SMDs <0.10 except for CA (SMD = 0.12), which remained within the acceptable range (<0.20) (Table 1). When IPCW was additionally applied, baseline balance was also maintained (Supplementary Table S10).

**Table 1:** Baseline characteristics of cohort participants according to dietary pattern, before and after inverse probability of treatment weighting (IPTW).

Characteristics	Unweighted (N = 71,579)			IPTW-Weighted (Σw = 69,659)^╪^				
	Low-risk dietary pattern (*N* = 24,256)	Moderate-risk dietary pattern (*N* = 23,703)	High-risk dietary pattern (*N* = 23,620)	Max pairwise SMD*	Low-risk dietary pattern (Σw = 23,420; rESS = 70.8%)	Moderate-risk dietary pattern (Σw = 23,676; rESS = 95.4%)	High-risk dietary pattern (Σw = 22,563; rESS = 64.4%)	Max pairwise SMD*
Variables used for IPTW calculation								
Age, years [mean (SE)]	45.47 (0.08)	40.56 (0.07)	34.59 (0.06)	1.01	40.88 (0.09)	40.26 (0.09)	39.50 (0.12)	0.12
Male, %	47.18	55.09	61.93	0.30	54.07	54.74	55.63	0.03
Han ethnicity, %	96.79	96.09	94.92	0.09	95.98	95.96	95.77	0.01
Married, %	93.01	86.64	73.18	0.53	76.41	75.97	76.08	0.05
Education >12 y, %	71.69	71.87	76.16	0.10	73.08	73.14	73.45	0.01
Alcohol use, %	19.59	29.98	41.63	0.48	28.69	30.36	31.20	0.06
Smoking, %	15.09	25.19	33.87	0.44	23.41	24.63	25.59	0.05
Adequate physical activities, %	47.30	31.73	18.37	0.62	33.82	32.66	29.62	0.09
Employment-based medical insurance, %	77.99	75.51	74.80	0.08	76.41	75.97	76.08	0.01
Diabetes, %	2.77	2.11	0.90	0.14	2.10	1.93	1.80	0.02
Hypertension, %	8.18	5.84	2.89	0.23	5.93	5.63	5.28	0.03
Variables not used for IPTW calculation								
KDM_BA at baseline^┼^, years [mean (SE)]	46.23 (0.08)	40.90 (0.08)	34.6 (0.07)	—	41.68 (0.09)	40.68 (0.09)	39.56 (0.12)	—
Hunan center^╫^, %	87.67	87.33	87.31	0.01	87.44	87.35	87.27	0.01

*Max pairwise SMD indicates the largest absolute SMD among the three dietary pattern groups (SMD < 0.10 good balance; 0.10–0.20 acceptable).

╪Weighted columns use stabilized IPTW with 1st–99th percentile truncation; IPCW is not applied in this table.

┼KDM-BA at baseline was not included in the IPTW model and is shown descriptively without SMD.

╫Study center was not included in the IPTW model but SMDs were presented to describe its distribution across dietary pattern groups.

IPTW, inverse probability of treatment weighting; SMD, standardized mean difference; Σw, sum of weights; rESS, relative effective sample size; SE, standard error; KDM: Klemera and Doubal’s method; BA: biological age.

### Dietary behavior pattern and change in biological age

At 12 months of follow-up, higher dietary behavior pattern risk was associated with greater increases in Δ*KDM-BA*, whereas no statistically significant associations were observed at 24 months. When dietary pattern was modeled as an ordinal exposure, per level increase in dietary risk was associated with 0.017 years higher Δ*KDM-BA* at 12 months (95% CI: 0.003, 0.030) (Table 2). In contrast, the corresponding association at 24 months was not statistically significant (*β* = 0.015, 95% CI: −0.046, 0.075). When dietary behavior pattern was modeled as a nominal exposure, similar patterns were observed, with stronger associations at 12 months but attenuation at 24 months. Compared with the low-risk group, the moderate- and high- risk groups showed 0.046 years (95% CI: 0.022, 0.071) and 0.034 years (95% CI: 0.006, 0.061) higher Δ*KDM-BA* at 12 months, and no significant associations were observed at 24 months (*p* > 0.05).

**Table 2:** Year-specific associations of dietary pattern with change in biological age (ΔKDM-BA) at 12- and 24- months.

Exposure	ΔKDM-BA at 12 months*			ΔKDM-BA at 24 months*			*P* for dietary pattern × time^┼^
	*β* (years)	95% CI	*p*-value	*β* (years)	95% CI	*p*-value	
Dietary pattern risk level as ordinal exposure^╪^							
Per-level increase in dietary pattern risk^╪^	0.017	(0.003, 0.030)	0.016	0.015	(−0.046, 0.075)	0.637	0.939
Dietary pattern as categorical variable							
Low-risk dietary pattern	Reference			Reference			0.720
Moderate-risk dietary pattern	0.046	(0.022, 0.071)	<0.001	0.005	(−0.104, 0.115)	0.924	
High-risk dietary pattern	0.034	(0.006, 0.061)	0.016	0.029	(−0.091, 0.149)	0.640	

*Weighted GEE model including both 12-month and 24-month time points with Gaussian identity link was used for association estimation and the final analysis weights = stabilized IPTW × year-specific IPCW weight; Model was adjusted for chronological age at baseline and study center. *β* > 0 indicates higher BA at follow-up compared to baseline (years).

╪Ordinal exposure treats dietary pattern as 0 = low-risk, 1 = moderate-risk and 2 = high-risk; Per-level increase indicates 0 increases to 1; or 1 to 2.

┼*P* for interaction was estimated through the Type-III Wald test.

KDM, Klemera and Doubal method; BA, biological age; ΔKDM-BA: change in KDM-BA at follow up compared to baseline KDM-BA; CA, chronological age.

In exploratory analyses using PCA-derived dietary factors, all three behavioral dimensions—irregular/discretionary & salty–eating-out, low-quality diet, and high-fat animal-food preference factors were positively associated with 12-month increases in Δ*KDM-BA* (*p* < 0.05), with the largest effect observed for the high-fat animal-food preference factor (Supplementary Table S11). However, associations at 24 months were not consistent in direction.

### Interactions of dietary behavior pattern with time and with chronological age

Regardless of whether dietary behavior pattern was modeled as an ordinal or nominal exposure, the interaction between time and dietary pattern risk was not statistically significant (*p* > 0.05), indicating no statistically detectable difference between the 12- and 24-month time points (Table 2).

Although the dietary behavior pattern effect appeared attenuated among older individuals (estimated per-level dietary behavior risk associated with 0.023 years higher Δ*KDM-BA* at age 30, 0.016 years at 40, and 0.007 years at 55), the interaction between dietary pattern and CA at baseline was not statistically significant (*p* = 0.656), suggesting no detectable effect modification by age (Table 3). Similar findings were obtained with dietary pattern assigned as a nominal exposure (Table 3).

**Table 3:** Interaction between chronological age at baseline and dietary pattern on the change in biological age (ΔKDM-BA)*.

Exposure	CA (years)	ΔKDM-BA, *β* (years) (95% CI)^₤^	*p*-value
Dietary pattern risk level as ordinal variable^╪^			
Per-level increase in dietary pattern risk × CA (per 10 y)^╪^	—	−0.007 (−0.035, 0.022)	0.656
Per-level increase in dietary pattern risk at representative age^┼^	30	0.023 (−0.015, 0.061)	0.239
	40	0.016 (−0.015, 0.047)	0.301
	55	0.007 (−0.051, 0.064)	0.825
Dietary pattern as categorical (reference = low-risk)			
Joint test for dietary pattern × CA^╫^	—		0.899
Moderate-risk dietary pattern × CA (per 10 y)	—	−0.005 (−0.051, 0.040)	0.815
High-risk dietary pattern × CA (per 10 y)	—	−0.014 (−0.072, 0.045)	0.648
Moderate vs. low-risk dietary pattern at representative age^┼^	30	0.052 (−0.006, 0.111)	0.079
	40	0.047 (0.021, 0.073)	<0.001
	55	0.039 (−0.026, 0.104)	0.236
High vs. low-risk dietary pattern at representative age^┼^	30	0.049 (−0.019, 0.116)	0.156
	40	0.035 (0.007, 0.063)	0.014
	55	0.015 (−0.074, 0.104)	0.747

*Combined GEE model including both 12-month and 24-month time points and the final analysis weights = stabilized IPTW × year-specific IPCW; age modeled continuously and centered at 40 and scaled per 10 years: CA’ = (CA − 40)/10.

₤Model was additional adjusted for centered chronological age at baseline and study center.

╪Dietary pattern treat as ordinal exposure: 0 = low-risk, 1 = moderate-risk and 2 = high-risk; Per-level increase indicates 0 changed to 1; or 1 changed to 2.

┼Estimates at 30/40/55 years are simple effects derived from the dietary pattern × age interaction model. *β* > 0 indicates higher BA at follow-up compared to baseline (years).

╫Joint test for interaction was estimated through the Type-III Wald tests.

KDM, Klemera and Doubal method; BA, biological age; ΔKDM-BA: change in KDM-BA; CA, chronological age.

### Sensitivity analysis

To attenuate potential bias from medication-related influences on BA, we excluded subjects with history of diabetes and hypertension for sensitivity analysis. Association between dietary patterns and Δ*KDM-BA* remained consistent with the primary analysis (Supplementary Table S12).

## Discussion

In this longitudinal cohort conducted in Hunan, China, we identified three dietary behavior patterns and found that individuals with higher-risk patterns experienced modest but detectable acceleration in the pace of biological aging over 12 months, with attenuated associations at 24 months. Component-level analyses further indicated that multiple behavioral dimensions contributed to these pattern-level associations. We observed no evidence of effect modification by chronological age.

Dietary behaviors can influence aging processes through multiple biological pathways, including metabolic regulation (24), inflammatory responses (25), and circadian rhythm disruption (26). Previous studies have reported associations between individual dietary habits, including irregular meal timing, preference for salty foods, regular consumption of sugar-sweetened beverages, and frequent fast-food intake, and accelerated aging or aging-related outcomes (15–17). Our findings are broadly consistent with the existing literature and further extend it by showing that multiple adverse dietary behaviors cluster within individuals and jointly contribute to short-term acceleration in the pace of biological aging, rather than operating in isolation.

Prior longitudinal analyses of Mediterranean-style dietary patterns and epigenetic aging indicate that structured dietary composition can slow biological aging, with effects that are most evident early and tend to weaken over time (27). Conceptualizing diet from a behavioral perspective, our study yields a comparable temporal pattern, indicating that the biological impact of adverse dietary behaviors likewise diminishes across follow-up. This time-dependent pattern likely reflects physiological adaptation to naturally occurring dietary behaviors. Even when dietary behaviors remain relatively stable, their physiological consequences may change over time, with early metabolic and inflammatory perturbations followed by compensatory responses that reduce the marginal impact of continued exposure. In contrast, the CALERIE trial implementing dynamic caloric restriction reports more persistent effects on biological aging (28), likely because its intervention strategies are continuously adjusted to counteract physiological compensation rather than reflecting fixed habitual exposure patterns. Importantly, the observed attenuation in our study does not contradict evidence linking dietary behaviors to long-term outcomes such as mortality. Short-term changes in biological aging primarily reflect dynamic and potentially reversible physiological responses, whereas long-term outcomes capture the cumulative burden of sustained exposure and progressive, largely irreversible structural damage. In this context, short-term changes in biological aging may be particularly informative as early indicators of physiological response rather than as surrogates for long-term structural damage.

Dietary studies in the Chinese population have revealed differences in eating habits among people born in different generations (29). In our study, younger individuals were more likely to exhibit higher-risk dietary behavior patterns, reflecting contemporary shifts in dietary habits. However, we observed no evidence of effect modification by chronological age in the association between dietary behavior patterns and short-term changes in the pace of biological aging. Several factors may explain the absence of detectable age-related heterogeneity. The biological pathways through which adverse dietary behaviors influence aging, such as metabolic dysregulation, low-grade inflammation, and altered lipid and glucose homeostasis, are operative throughout adulthood and may exert comparable short-term effects on aging pace regardless of age (30). In addition, our analyses focused on short-term changes in aging pace rather than long-term cumulative aging or disease outcomes, for which age-related differences may be more pronounced. In this context, chronological age likely influences baseline levels of biological aging, whereas short-term rates of change in aging remain relatively stable across age groups. From a public health perspective, these findings underscore that unhealthy dietary behavior patterns are relevant across the adult life course. Although younger adults were more likely to adopt higher-risk dietary behaviors, dietary behavior interventions may confer physiological benefits at multiple stages of adulthood rather than being limited to older populations.

This study has several important strengths. The large sample size and longitudinal design enabled us to examine short-term changes in the pace of biological aging in relation to dietary behavior patterns within individuals. By characterizing dietary exposure at the pattern level using latent class analysis, complemented by exploratory component-level analyses, we captured the co-occurrence of multiple eating-related behaviors that may not be adequately reflected by conventional nutrient- or food-based measures. In addition, the application of inverse probability weighting to balance baseline characteristics and to account for selective follow-up enhanced the robustness of our estimates in this observational setting.

Several limitations should also be acknowledged when interpreting findings. First, participants were not selected through population-based random sampling. Although the study included participants from both southern and northern China, it comprised hospital-based health check-up participants predominantly from urban and peri-urban areas, with limited representation of rural populations. Therefore, the generalizability of our findings, particularly to rural populations, requires further evaluation. As with all observational studies, residual confounding cannot be fully excluded, even with extensive adjustment and weighting. Dietary behaviors were assessed at baseline only, and changes in behaviors during follow-up were not captured, which may have contributed to the attenuation of associations over time and limited our ability to evaluate the effects of sustained behavioral change. Follow-up at 24 months was less complete than at 12 months, which may have reduced statistical precision, although censoring weights were applied to mitigate potential selection bias. Finally, the biological aging measure used in this study was derived primarily from physiological biomarkers and is likely more sensitive to short-term, potentially reversible changes than to cumulative structural organ damage. Future studies incorporating repeated assessment of dietary behaviors and complementary long-term functional or clinical outcomes will be important to further clarify the long-term health implications of dietary behavior patterns.

## Conclusion

In this longitudinal cohort study, we found that unhealthy dietary behaviors were associated with short-term increases in the pace of biological aging, although these associations appeared to attenuate over time, potentially reflecting physiological adaptation. Our results support the use of biological aging, particularly short-term changes in aging pace, as a meaningful intermediate indicator for capturing early physiological responses to dietary behaviors. From a clinical and public health perspective, maintaining healthy dietary behaviors may require continuous monitoring and adaptation rather than one-time interventions. Further intervention studies are needed to determine whether changes in biological aging measures translate into long-term health benefits.

## Data Availability

The data analyzed in this study is subject to the following licenses/restrictions: the datasets used and analyzed during the current study are available from the corresponding author on reasonable request. Requests to access these datasets should be directed to YL, lydia0312_2000@163.com.

## Footnotes

1
https://github.com/dayoonkwon/BioAge
