# Supplementary material for: Dietary behavior patterns and short-term pace of aging—evidence from a Chinese cohort
Source: Front Nutr. 2026 Sep 9;13:1924515. doi: 10.3389/fnut.2026.1924515 (PMC13597222; doi:10.3389/fnut.2026.1924515)
Supplement: Supplementary file 1 [file Supplementary_file_1.docx]

Supplementary Material

Supplementary Methods S1. Derivation and validation of the KDM-based biological age index

Supplementary Methods S2. Derivation of dietary behavior patterns using PCA and LCA

Supplementary Methods S3. Inverse probability weighting and statistical modeling

Table S1. Pearson’s correlation coefficients between CA and organ system related biomarkers or physical measurements among cross-sectional population (n=160,916)

Table S2. Variance inflation factor (VIF) results of different biomarker combinations for collinearity assessment in the Cross sectional population (n=160,916)

Table S3. Model performance of KDM-BA constructed using different biomarker combinations in the cross-sectional population (n=160,916)

Table S4. Correlation and agreement between KDM-BA and chronological age (CA) in the cross-sectional and cohort population

Figure S1 Scree plot of Eigenvalues for factor analysis on dietary behaviors

Figure S2 The association Pearson correlation between KDM-BA at baseline and CA among cohort population (n=71,579)

Table S5. Questionnaire of dietary habits

Table S6. Rotated factor pattern of 23 dietary behavior variables (N=160,916)

Table S7. Inter-factor correlations (Promax rotation)

Table S8. Model fit statistics and class proportions for latent class analysis (LCA) of dietary behavior patterns

Table S9. Item-response probabilities and class membership for the three dietary behavior patterns derived from latent class analysis (LCA)

Table S10. Baseline characteristics of analytic samples used for the 12-month and 24-month biological age increment analyses, before and after applying final stabilized analysis weights (IPTW × IPCW)

Table S11. Associations between PCA derived dietary factors and ΔKDM-BA at 12- and 24-months

Table S12. Sensitive analysis on the associations of dietary pattern with change in biological age (ΔKDM-BA) at 12- and 24- months and interactions with time among population without hypertension or diabetes mellitus (n= 66,446)

Supplementary Methods S1. Derivation and validation of the KDM-based biological age index

Biological age was derived using the Klemera–Doubal method (KDM), which estimates an individual’s biological aging status by integrating information from multiple physiological biomarkers. The derivation procedure followed previously published algorithms and reference implementations, with adaptations to the available biomarker panel in the present dataset.

Using the cross-sectional dataset, candidate biomarkers spanning hepatic, renal, metabolic, hematologic/immune, cardiovascular, and anthropometric domains were first screened for their associations with chronological age (CA). Sex-specific Pearson correlation coefficients between each biomarker and CA were calculated. Biomarkers with an absolute correlation coefficient |r| > 0.10 in both sexes were included in the initial candidate pool, and additional biomarkers with |r| > 0.10 in either sex were subsequently evaluated.

Multiple candidate biomarker combinations were constructed and assessed for multi-collinearity using variance inflation factors (VIFs). Combinations with any VIF ≥ 5 were excluded. Among the remaining candidate models, selection was based on overall performance metrics, including (i) correlation between KDM-based biological age and CA, (ii) root mean square error (RMSE), and (iii) calibration slope from linear regression of biological age on CA. The final model was selected to achieve strong correlation with CA (r > 0.80 in both sexes), low RMSE, and satisfactory calibration (slope approximately equal to 1).

The final KDM-based biological age model included 14 biomarkers representing multiple physiological systems: albumin (ALB), blood urea nitrogen (BUN), total cholesterol (TC), glycated hemoglobin (HbA1c), mean corpuscular volume (MCV), platelet count (PLT), systolic blood pressure (SBP), diastolic blood pressure (DBP), body roundness index (BRI), waist-to-hip ratio (WHR), resting heart rate (HR), serum creatinine (SCr), and total protein (TP).

Model performance was evaluated separately in men and women. Detailed performance metrics, including correlations with CA, RMSE, and calibration plots, are presented in Supplementary Tables S1– S4. The derived model was subsequently applied to the longitudinal cohort.

In the longitudinal dataset, missing biomarker values at baseline were imputed using age- and sex-specific means. Missing biomarker values at follow-up visits were imputed using last observation carried forward (LOCF) at the individual level. Biological age was calculated at baseline and follow-up examinations using the fixed parameters derived from the cross-sectional dataset. Short-term change in biological age was defined as the difference between follow-up and baseline biological age values.

All procedures for biological age derivation were implemented in R (version 4.4.3)

Supplementary Methods S2. Derivation of dietary behavior patterns using PCA and LCA

Dietary behavior patterns were derived using a two-stage approach combining principal component analysis (PCA) and latent class analysis (LCA). Binary dietary behavior variables were coded as 0/1 before PCA. PCA with oblique (Promax) rotation was first applied to the 23 dietary behavior variables to identify correlated behavioral dimensions. The number of retained factors was determined based on eigenvalues (>1), scree plot inspection, and interpretability of factor loadings. Factor loadings ≥0.30 were considered meaningful for factor interpretation.

Factor scores from the retained PCA dimensions were standardized and categorized into tertiles. These categorized factor scores were then used as indicators in LCA to identify discrete dietary behavior patterns. Model selection was based on BIC, adjusted BIC, entropy, average posterior probabilities, and substantive interpretability. Models with increasing numbers of classes were evaluated using the Bayesian Information Criterion (BIC), entropy, and class interpretability. A three-class solution was selected based on optimal model fit and substantive interpretability.

To apply the derived dietary behavior patterns to the longitudinal cohort, posterior class probabilities were calculated using fixed LCA parameters estimated in the cross-sectional dataset. Tertile cut-offs were derived from the cross-sectional dataset and consistently applied to the longitudinal cohort. Each participant was assigned to the dietary behavior pattern with the highest posterior probability.

Supplementary Methods S3. Inverse probability weighting and statistical modeling

Stabilized inverse probability of treatment weights (IPTW) were estimated using a multinomial logistic regression model with dietary behavior pattern as the outcome variable. Dietary behavior pattern was coded as 1 (low-risk, reference), 2 (moderate-risk), and 3 (high-risk). Baseline covariates included chronological age, sex, ethnicity, education, marital status, smoking status, alcohol consumption, adequate physical activity, employment-based medical insurance, history of hypertension, and history of diabetes.

Let Aᵢ denote the dietary behavior pattern of participant i and Xᵢ denote baseline covariates. The stabilized IPTW was calculated as:

*SWᵢᴵᴾᵀᵂ* = *P(Aᵢ*=*a) / P(Aᵢ*=*a* | *Xᵢ)*

Covariates were selected a priori based on their potential associations with dietary behavior patterns and biological aging. Continuous age was entered without categorization, whereas categorical variables were coded as indicated below.

| Variable | Coding | Role in weighting models |
| --- | --- | --- |
| Dietary behavior pattern | 1=Low-risk (reference); 2=Moderate-risk; 3=High-risk | Outcome in IPTW; covariate in IPCW |
| Availability of 12-month BA measurement | 0=No; 1=Yes | Outcome in 12-month IPCW |
| Availability of 24-month BA measurement | 0=No; 1=Yes | Outcome in 24-month IPCW |
| Chronological age | Continuous, years | Covariate |
| Sex | Female=0; Male=1 | Covariate |
| Ethnicity | Non-Han=0; Han=1 | Covariate |
| Education | ≤12 years=0; >12 years=1 | Covariate |
| Marital status | Married=0; Not married=1 | Covariate |
| Smoking | No=0; Yes=1 | Covariate |
| Alcohol consumption | No=0; Yes=1 | Covariate |
| Physical activity | Adequate=0; Inadequate=1 | Covariate |
| Employment-based medical insurance | No=0; Yes=1 | Covariate |
| Hypertension | No=0; Yes=1 | Covariate |
| Diabetes mellitus | No=0; Yes=1 | Covariate |

Stabilized inverse probability of censoring weights (IPCW) were estimated separately for the 12-month and 24-month follow-up windows using pooled logistic regression models in the baseline-eligible population, regardless of follow-up completion. Dietary behavior pattern was included as a covariate in IPCW models because follow-up availability may differ according to baseline dietary behavior pattern. Let *SW_i,t_ ^IPCW^* denote the stabilized IPCW for individual *i* at follow-up time *t* (12 or 24 months), relative to baseline, defined as

*SW_i,t_ ^IPCW^* = *P(R_i,t_*=*1) / P(R_i,t_*=*1* | *Aᵢ, Xᵢ)*

Final analysis weights were defined as the product of stabilized inverse probability of treatment weights (IPTW) and time-specific inverse probability of censoring weights (IPCW) and were truncated at the 1st and 99th percentiles to reduce the influence of extreme weights, such that

*Wᵢ_,t_* = *SW_i_ ᴵᴾᵀᵂ*× *SW_i,t_ ^IPCW^*

Weighted generalized estimating equation (GEE) models were used to estimate associations between dietary behavior patterns and short-term changes in the pace of aging, operationalized as changes in biological age (*ΔKDM-BA*), across follow-up windows.

Let *ΔKDM-BA_i,t_* denote the change in biological age for individual *i* at follow-up time *t* (12 or 24 months), relative to baseline, defined as

*ΔKDM-BAᵢ,ₜ* = *KDM-BAᵢ,ₜ* − *KDM-BAᵢ,₀*

The primary weighted generalized estimating equation (GEE) model was specified as:

*ΔKDM-BAᵢ,ₜ = β₀ + β₁g(Aᵢ) + β₂Tₜ + β₃[g(Aᵢ)×Tₜ] + β₄CAᵢ^’^ + β₅[g(Aᵢ)×CAᵢ^’^] + εᵢ,ₜ*

where *Aᵢ* denotes dietary behavior pattern, *g*(*A*ᵢ) denotes dietary pattern coding, Tₜ denotes follow-up time (12 vs. 24 months), and *CAᵢ* denotes baseline chronological age centered at 40 years and scaled per 10-year increment (*CA_i_^′^* = (*CA_i_*−40)/10)).Dietary pattern was modeled in two ways. First, it was coded as an ordinal exposure with values *g*(*A*_i_)∈{0, 1, 2}, corresponding to low-, moderate-, and high-risk dietary behavior patterns, respectively, allowing estimation of per-level increases in dietary pattern risk. Second, dietary behavior pattern was modeled as a nominal exposure using indicator variables for moderate-risk and high-risk patterns, with the low-risk pattern serving as the reference group.

Stabilized inverse probability of treatment and censoring weights were applied in all models. Weighted GEE models with an identity link and Gaussian variance were fitted, accounting for within-individual correlation across follow-up time points. Type III Wald tests were used to assess interaction terms between dietary behavior pattern and follow-up time, as well as between dietary behavior pattern and baseline chronological age.

Simple effects of dietary behavior pattern on changes in biological age were estimated at each follow-up time point and at representative ages (30, 40, and 55 years), based on the fitted interaction models.

To explore which components contributed to the pattern associations, the three PCA-derived factors (in tertiles) were analyzed using IPCW only GEE models adjusted for baseline covariates. We also re-estimated the primary models after excluding participants with history of hypertension or diabetes at baseline.

Table S1. Pearson’s correlation coefficients between CA and organ system related biomarkers or physical measurements among cross-sectional population (n=160,916)

| Organ system | Variables | Correlation with chronological age (r) | | |
| --- | --- | --- | --- | --- |
|  |  | Overall  (n=160,916) | Male  (n=86,035) | Female  (n=74,881) |
| Hepatic | Albumin, g/dL | **-0.355*** | **-0.449*** | **-0.280*** |
|  | Total protein, g/dL | **-0.108*** | **-0.165*** | -0.043* |
| Renal | ln(Creatinine), mg/dL | 0.066* | 0.004 | **0.143*** |
|  | ln(SUA), mg/dL | 0.002 | **-0.121*** | 0.098* |
|  | ln(BUN), mg/dL | **0.267*** | **0.215**** | **0.324*** |
| Metabolic | Total cholesterol, mg/dL | **0.279*** | **0.144** | **0.424*** |
|  | HDL_c, mg/dL | 0.039* | **0.111*** | 0.002 |
|  | ln(HbA1c), % | **0.292*** | **0.264*** | **0.345*** |
| Immune-Inflammatory | WBC, ×10⁹/L | -0.052* | -0.024* | -0.099* |
|  | Lymphocyte % | -0.099* | **-0.177*** | -0.013* |
|  | Monocyte % | 0.026* | 0.036* | 0.005* |
| Hematologic | Platelets, ×10⁹/L | **-0.175*** | **-0.201*** | **-0.144*** |
|  | RBC ×10^12/^L | **-0.142*** | **-0.313*** | -0.054* |
|  | MCV, fL | **0.166*** | **0.211*** | **0.116*** |
|  | Hemoglobin, g/dL | -0.063* | **-0.234*** | 0.013* |
| Cardiovascular | SBP, mmHg | **0.350*** | **0.256*** | **0.461*** |
|  | DBP, mmHg | **0.255*** | **0.242*** | **0.284*** |
|  | Heart rate, times/min | **-0.155*** | -0.090* | **-0.223*** |
| Adiposity/ anthropometric | Waist circumference, cm | **0.255*** | **0.144*** | **0.454*** |
|  | Waist-to-hip ratio | **0.375*** | **0.326*** | **0.527*** |
|  | Body Round Index^┼^ | **0.383*** | **0.270*** | **0.526*** |
|  | BMI, kg/m^2^ | **0.177*** | 0.050* | **0.339*** |

* P-value<0.0001;

^┼^ BRI = 364.2–365.5 × (1 − ((0.5 × WC/π)^2^/(0.5 × Ht)^2^))^0.5^

Abbreviations: CA, chronological age; ln, natural logarithm; SUA, serum uric acid; BUN, blood urea nitrogen; HDL_c, high density lipoprotein cholesterol; LDL_c, low density lipoprotein cholesterol; Hba1c, glycated hemoglobin A1c; WBC, white blood cell; RBC, red blood cell; LYM%, lymphocyte percentage; MCV, mean corpuscular volume; SBP, systolic blood pressure; DBP, diastolic blood pressure; BMI, body mass index; WC, waist circumstance; Ht, height.

Table S2. Variance inflation factor (VIF) results of different biomarker combinations for collinearity assessment in the Cross sectional population (n=160,916)

| Combination | No. of indices | Variables | Max VIF (in male) | Max VIF (in female) | No. of systems represented | Remarks |
| --- | --- | --- | --- | --- | --- | --- |
| 1 | 11 | Alb, BUN,TC, HbA1c, MCV, Plt, SBP, DBP, WC, BRI, WHR | 9.55 | 12.2 | 6 | Excluded (VIF ≥ 5), multi-collinearity among BRI and waist |
| 2 | 10 | Alb, BUN,TC, HbA1c, MCV, Plt, SBP, DBP, WC, WHR | 3.31 | 3.64 | 6 | Acceptable |
| 3 | 10 | Alb, BUN,TC, HbA1c, MCV, Plt, SBP, DBP, BRI, WHR | 3.62 | 4.10 | 6 | Acceptable |
| 4 | 11 | Combination 2/3 + BMI | 10.40 | 11.60 | 6 | Excluded (VIF ≥ 5), multi-collinearity among BMI, WC, and BRI |
| 5 | 12 | Combination 2/3 + RBC, Hb | 9.92 | 11.20 | 6 | Excluded (VIF ≥ 5), multi-collinearity among MCV, RBC and Hb |
| 6* | 17 | Combination 2/3 + TP, Scr, SUA, HDL_c, LYM%, RBC, HR | 3.79 | 4.28 | 7 | Acceptable |
| 7* | 17 | Combination 2/3 + TP, Scr, SUA, HDL_c, LYM%, Hb, HR | 3.79 | 4.27 | 7 | Acceptable |

* Combinations 6 and 7 showed acceptable multi-collinearity (all VIFs < 5) in both sexes. The final KDM-BA model was derived by further reducing several variables from these combinations, and all variables in the final model also had VIF < 5 (see Supplementary Table 3).

Abbreviations: Alb, Albumin; BUN, blood urea nitrogen; TC, total cholesterol; HbA1c, glycated hemoglobin A1c; MCV, mean corpuscular volume; Plt, platelets; SBP, systolic blood pressure; DBP, diastolic blood pressure; WC, waist circumstance; WHR, Waist-to-hip ratio; BRI, body round index; BMI, body mass index; SUA, serum uric acid; HDL_c, high density lipoprotein cholesterol; RBC, red blood cell; LYM%, lymphocyte percentage.

Table S3. Model performance of KDM-BA constructed using different biomarker combinations in the cross-sectional population (n=160,916)

| Model | No. of indices | No. of systems represented | Variables | Male (n=86,035) | | | Female (n=74,881) | | |
| --- | --- | --- | --- | --- | --- | --- | --- | --- | --- |
|  |  |  |  | r | RMSE | β (95%CI)* | r | RMSE | β (95%CI)* |
| 1 | 10 | 6 | Alb, BUN,TC, Hba1c, MCV, Plt, SBP, DBP, WC, WHR | 0.462 | 23.90 | 1.000 (0.987–1.013) | 0.994 | 1.36 | 1.000 (0.999–1.001) |
| 2 | 10 | 6 | Alb, BUN,TC, Hba1c, MCV, Plt, SBP, DBP, BRI, WHR | 0.576 | 17.80 | 1.006 (0.996–1.015) | 0.999 | 0.51 | 1.000 (1.000–1.001) |
| 3 | 11 | 7 | Model 2 + LYM% | 0.574 | 17.90 | 1.006 (0.996–1.015) | 0.994 | 1.41 | 0.999 (0.998–1.000) |
| 4 | 11 | 6 | Model 2 + Scr | 0.836 | 8.19 | 1.003 (0.998–1.007) | 1.000 | 0.99 | 1.000 (1.000–1.000) |
| 5 | 11 | 6 | Model 2 + HR | 0.650 | 14.60 | 1.005 (0.997–1.013) | 0.995 | 1.31 | 1.001 (1.000–1.002) |
| 6 | 11 | 6 | Model 2 + RBC | 0.491 | 22.20 | 1.007 (0.995–1.019) | 0.998 | 0.75 | 0.999 (0.999–1.000) |
| 7 | 11 | 6 | Model 2 + TP | 0.622 | 15.70 | 1.005 (0.996–1.013) | 0.997 | 0.99 | 0.999 (0.999–1.000) |
| 8 | 11 | 6 | Model 2 + SUA | 0.583 | 17.40 | 1.006 (0.996–1.015) | 0.996 | 1.15 | 0.999 (0.998–1.000) |
| 9 | 11 | 6 | Model 2 + HDL_c | 0.568 | 18.10 | 1.006 (0.996–1.016) | 0.993 | 1.52 | 0.999 (0.998–1.000) |
| 10 | 12 | 6 | Model 2 + Scr, HR | 0.865 | 7.24 | 1.002 (0.998–1.006) | 0.998 | 0.71 | 1.001 (1.000–1.001) |
| 11 | 12 | 6 | Model 2 + Scr, TP | 0.851 | 7.70 | 1.002 (0.998–1.007) | 0.995 | 1.28 | 0.999 (0.998–1.000) |
| **12** | **13** | **6** | **Model 2 + Scr, HR, TP** | **0.879** | **6.76** | **1.002 (0.998–1.006)** | **0.999** | **0.50** | **1.000 (****0.999–1.000)** |
| 13 | 13 | 6 | Model 1+ Scr, HR, TP | 0.794 | 9.53 | 1.000 (0.995–1.005) | 1.000 | 0.20 | 1.000 (1.000–1.000) |
| 14 | 14 | 7 | Model 2 + Scr, HR, TP, LYM% | 0.857 | 7.52 | 1.002 (0.998–1.006) | 0.991 | 1.67 | 0.999 (0.998–1.000) |
| 15 | 14 | 6 | Model 2 + Scr, HR, TP, SUA, | 0.861 | 7.93 | 1.002 (0.998–1.006) | 0.992 | 1.57 | 0.999 (0.998–1.000) |
| 16 | 14 | 6 | Model 2 + Scr, HR, TP, HDL_c, | 0.848 | 7.81 | 1.002 (0.998–1.007) | 0.991 | 1.75 | 0.999 (0.998–1.000) |
| 17 | 14 | 6 | Model 2 + Scr, HR, TP, RBC | 0.834 | 8.27 | 1.002 (0.998–1.007) | 0.996 | 1.18 | 0.999 (0.998–1.000) |
| 18 | 17 | 7 | Model 2 + Scr, HR, TP, SUA, HDL_c, LYM%, RBC | 0.801 | 9.33 | 1.003 (0.998–1.008) | 0.977 | 2.78 | 0.998 (0.996–0.999) |

* Calibration slope (β) was estimated from linear regression of KDM-BA on chronological age (CA) within each sex stratum; β values close to 1 indicate better calibration. Models with higher r, lower RMSE, and β≈1 were considered to have better overall performance.

The final KDM-BA model 12 was derived from the acceptable combinations 6 by further reducing several variables, and all variables in the final model had VIF < 5 (see Supplementary Table 3).

Abbreviations: KDM: Klemera and Doubal’s method; BA: biological age; CA: chronological age; RMSE: root mean square error; Alb, Albumin; BUN, blood urea nitrogen; TC, total cholesterol; HbA1c, glycated hemoglobin A1c; MCV, mean corpuscular volume; Plt, platelets; SBP, systolic blood pressure; DBP, diastolic blood pressure; WC, waist circumstance; WHR, Waist-to-hip ratio; BRI, body round index; BMI, body mass index; SUA, serum uric acid; HDL_c, high density lipoprotein cholesterol; RBC, red blood cell; LYM%, lymphocyte percentage.

Table S4. Correlation and agreement between KDM-BA and chronological age (CA) in the cross-sectional and cohort population*

|  | Cross-sectional population  (n=160,916) | | | Cohort population  (n=71,579) | | |
| --- | --- | --- | --- | --- | --- | --- |
|  | Overall | Male | Female | Overall | Male | Female |
| CA, mean(sd), year | 42.29 (12.56) | 42.57 (12.45) | 41.96 (12.68) | 40.25 (11.84) | 41.63 (12.12) | 38.59 (11.27) |
| KDM-BA, mean(sd), year | 42.31 (13.51) | 42.63 (14.19) | 41.95 (12.68) | 40.68 (12.83) | 42.46 (13.77) | 38.53 (11.22) |
| RMSE, year | 4.95 | 6.76 | 0.50 | 4.80 | 6.48 | 0.47 |
| Pearson’s correlation coefficient between KDM-BA and CA | 0.930 | 0.879 | 0.999 | 0.928 | 0.884 | 0.999 |

* KDM-BA estimation model comprised albumin, blood urea nitrogen, total cholesterol, glycated hemoglobin A1c, mean corpuscular volume, platelets, systolic blood pressure, diastolic blood pressure, waist-to-hip ratio, body round index, heart rate, serum creatinine, and total protein. Values are presented as mean (SD). Pearson’s r represents the correlation between KDM-BA and CA. KDM-BA performance was evaluated in both the cross-sectional population used for model training and an independent cohort population.

Abbreviations: KDM: Klemera and Doubal’s method; BA: biological age; CA: chronological age; RMSE: root mean square error.

**Figure S1 Scree plot of Eigenvalues for factor analysis on dietary behaviors**

**Figure S2 The association Pearson correlation between KDM-BA at baseline and CA among cohort population (n=71,579)**

Abbreviations: KDM: Klemera and Doubal’s method; CA: chronological age; BA: biological age;

**Table S5. Questionnaire of dietary habits ***

1. Are you usually able to have three meals on time?

A. Yes

B. Mostly

C. No

2. Do you often overeat?

A. Yes

B. No

3. Do you often eat late-night snacks?

A. Do not eat

B. Occasionally eat

C. Often eat

4. How often do you participate in meals (social gatherings)?

A. Rarely or occasionally (1-2 times/month)

B. Quite often (1-2 times/week)

C. Frequently (3-5 times/week)

D. Very frequently (more than 5 times/week)

5. What are your food preferences (multiple choice)? ^┼^

A. Light *

B. Salty

C. Sweet *

D. High-fat *

E. Spicy

F. Hot

6. What are your dietary preferences (multiple choice)? ^┼^

A. Smoked and pickled foods

B. Fried foods

C. Desserts *

D. Snacking (excluding moderate amounts of nuts)

E. Eating fast food

F. Drinking porridge (≥2 times/day) *

G. Other *

7. How is your structure of staple food?

A. Fine grain

B. Coarse grain and fine grain mix

C. Coarse grain

D. Hard to say

8. Do you drink milk (yogurt)?

A. Not at all

B. Occasionally (1-2 times/week)

C. Frequently (3-5 times/week)

D. Every day (more than 5 times/week)

9. Do you eat eggs?

A. Do not eat

B. Eat occasionally occasionally (1-2 times / week)

C. Eat regularly (3-5 times / week)

D. Eat every day (> 5 times / week)

10. Do you eat beans and soy products

A. Don't eat

B. Eat it occasionally (1-2 times / week)

C. Eat it regularly (3 times / week)

11. Do you eat fruit?

A. Not at all

B. Occasionally (1-2 times/week)

C. Frequently (3-5 times/week)

D. Every day (more than 5 times/week)

12. On average, how many vegetables do you eat per day?

A. <100g

B. 100-200g

C. 200-500g

D. >500g

13. How much meat do you eat on average (pork, beef, mutton, poultry) every day?

A. <50g

B. 50-100g

C. 101-250g

D. >250g

14. Do you eat fat?

A. Do not eat

B. Eat a little occasionally

C. Eat regularly

15. Do you eat the animal offal?

A. Do not eat

B. Eat occasionally (1-2 times / week)

C. Eat regularly (3 times / week)

16. Do you eat fish or seafood?

A. Do not eat

B. Eat occasionally (1-2 times / week)

C. Eat regularly (3 times / week)

17. Do you have coffee?

A. Do not drink

B. Drink occasionally (1-2 times / week)

C. Drink often (3-5 times / week)

D. Drink every day (> 5 times / week)

18. Do you drink sugary drinks (fruit juice, cola, etc.)?

A. Do not drink

B. Drink occasionally (1-2 times / week)

C. Drink often (3-5 times / week)

D. Drink every day (> 5 times / week)

* Table S5 presents the latest version of the 18-question online self-reported dietary questionnaire, revised in 2019. The questionnaire structure remained unchanged, but additional response options were added to Questions 5 and 6 in the revised version. Therefore, 23 dietary behavior variables were consistently available across the study period, with six additional variables available only for participants assessed using the revised questionnaire.

^┼^ Questions 5 and 6 allowed multiple responses, with each response option recorded as a separate binary (yes/no) variable. Thus, the 18 questionnaire questions yielded multiple dietary behavior variables for subsequent analyses.

| **Table S6. Rotated factor pattern of 23 dietary behavior variables (N=160,916) *** | | | |
| --- | --- | --- | --- |
| Dietary behavior variables | Factor1 | Factor2 | Factor3 |
| Often night eating | **0.49958** | 0.02108 | 0.12871 |
| Often skip meals | **0.45951** | 0.05407 | -0.12251 |
| Often drinking sweeten beverages | **0.39052** | -0.08450 | 0.00429 |
| Often eating fast foods | **0.37121** | -0.07913 | -0.03829 |
| Often over eating | **0.34956** | 0.10272 | -0.02985 |
| Often dinning out for business | 0.27234 | 0.04527 | 0.13403 |
| Often drinking coffee | 0.23166 | -0.19487 | -0.03378 |
| Often eating snacks | 0.19933 | -0.01899 | -0.07570 |
| Preference to salty diet | **0.33417** | 0.19121 | 0.17655 |
| Preference to fried food | 0.18715 | 0.05398 | 0.08577 |
| Preference to pickled food | 0.01550 | 0.18191 | 0.12592 |
| Preference to spicy food | 0.14181 | 0.13627 | 0.12257 |
| Preference to hot food | 0.03298 | 0.07014 | 0.07276 |
| Eating eggs <3 per week | -0.01370 | **0.46631** | -0.09741 |
| Drinking milk or yogurt <3 per week | -0.08233 | **0.45334** | 0.04679 |
| Eating fruits <3 per week | 0.04299 | **0.44296** | 0.03779 |
| Eating beans <3 per week | -0.08183 | **0.40284** | -0.07993 |
| Eating vegetables <100 grams per day | 0.15656 | 0.25405 | -0.08573 |
| Not eating coarse grains | 0.05311 | 0.18144 | 0.03079 |
| Not eating fish or seafood | 0.00504 | 0.10886 | **-0.33319** |
| Eating animal offal per week | -0.09948 | 0.03420 | **0.61287** |
| Eating fat meats per week | -0.08434 | 0.07130 | **0.45323** |
| Eating meats≥ 50 grams per day | 0.11856 | -0.10501 | **0.40062** |

* Promax rotation and factor loadings ≥0.30 are highlighted in bold; The results indicated that factor 1 represented discretionary/irregular eating & salty–eating-out pattern, factor 2 represented low-quality diet pattern and factor 3 represented high-fat animal foods preference pattern.

| **Table S7. Inter-factor correlations (Promax rotation)*** | | | |
| --- | --- | --- | --- |
|  | Factor1 | Factor2 | Factor3 |
| Factor1 | 1.00000 | 0.28923 | 0.29088 |
| Factor2 | 0.28923 | 1.00000 | 0.09289 |
| Factor3 | 0.29088 | 0.09289 | 1.00000 |

* Factor 1: Discretionary/irregular eating & salty–eating-out pattern; Factor 2: Low-quality diet; Factor 3: High-fat animal foods preference.

| **Table S8. Model fit statistics and class proportions for latent class analysis (LCA) of dietary behavior patterns（N=160,916）** | | | | | |
| --- | --- | --- | --- | --- | --- |
| Class | BIC | Adjusted BIC | Entropy | Average posterior probability | Estimated class membership probabilities (%) |
| 2 | 2154.88 | 2113.56 | 0.77 | 0.916/ 0.928 | 43.25 / 56.75 |
| **3*** | **329.66** | **266.10** | **0.76** | **0.999/ 0.942 /0.766** | **36.41/ 38.03/ 25.55** |
| 4 | 344.38 | 258.58 | 0.58 | 0.762/ 0.830/ 0.817 /0.736 | 25.82/ 16.12 / 35.42 / 22.64 |
| 5 | 407.63 | 299.57 | 0.54 | 0.817/0.552/0.685/0.604/0.870 | 31.19/ 14.78/ 9.54 / 18.39 / 26.09 |

* The three-class solution was selected based on the overall balance of model fit, classification quality, and substantive interpretability. It had the lowest BIC and higher entropy than the four- and five-class solutions, although the four-class solution had a slightly lower adjusted BIC, and acceptable entropy (0.76).

Abbreviation: BIC, Bayesian Information Criterion.

| **Table S9. Item-response probabilities and class membership for the three dietary behavior patterns derived from latent class analysis (LCA) （N=160,916）** | | | | | | |
| --- | --- | --- | --- | --- | --- | --- |
| Latent class^┼^ | | Response probabilities, % (SE) | | | Class size, % (SE) | |
|  |  | Low Category* | Medium Category* | High Category* | |  |
| Low-risk dietary pattern | | | | | 36.41 (0.51) | |
|  | Factor 1 ^┼^ | **91.25 (1.24)** | 5.29 (0.92) | 3.45 (0.81) | |  |
|  | Factor 2 ^┼^ | **50.97 (0.22)** | 33.59 (0.20) | 15.44 (0.16) | |  |
|  | Factor 3 ^┼^ | **53.69 (0.22)** | 37.63 (0.21) | 8.69 (0.14) | |  |
| Moderate-risk dietary pattern | | | | | 38.03 (1.96) | |
|  | Factor 1 ^┼^ | 0.06 (0.18) | **82.70 (4.17)** | 17.24 (4.18) | |  |
|  | Factor 2 ^┼^ | 27.17 (0.34) | 36.39 (0.22) | **36.44 (0.32)** | |  |
|  | Factor 3 ^┼^ | 26.64 (0.36) | 34.95 (0.22) | **38.41 (0.40**) | |  |
| High-risk dietary pattern | | | |  | | 25.55 (1.66) |
|  | Factor 1 ^┼^ | 0.02 (0.05) | 0.09 (0.28) | **99.89 (0.28)** | |  |
|  | Factor 2 ^┼^ | 17.35 (0.49) | 28.03 (0.58) | **54.61 (0.99)** | |  |
|  | Factor 3 ^┼^ | 14.16 (0.59) | 24.90 (0.66) | **60.94 (1.19)** | |  |

* Higher factor-category values indicate a greater likelihood of exhibiting the corresponding unhealthy dietary behavior.

^┼^ Factor 1: discretionary/irregular eating and salty–eating-out pattern; Factor 2: low-quality diet; Factor 3: high-fat animal-foods preference.

| **Table S10. Baseline characteristics of analytic samples used for the 12-month and 24-month biological age increment analyses, before and after applying final stabilized analysis weights (IPTW × IPCW)** | | | | | | | | | |  |
| --- | --- | --- | --- | --- | --- | --- | --- | --- | --- | --- |
| Characteristics | Unweighted | | | | Weighted (IPTW*IPCW)^╪^ | | | | |  |
|  | Low-risk dietary pattern | Moderate-risk dietary pattern | High-risk dietary pattern | Max pairwise SMD* | | Low-risk dietary pattern | Moderate-risk dietary pattern | High-risk dietary pattern | Max pairwise SMD* |  |
| 12-month analytic sample (Cohort participants with BA measured at 10–14 months, n= 50,603) | | | | | | | | | | |
|  | N=17,116 | N=16,787 | N=16,700 |  | | Σw =15,890, rESS=60.4% | Σw =16,517, rESS=79.7% | Σw =16,411, rESS=46.8% |  |  |
| Age, years (mean (SE)) | 45.52 (0.09) | 40.35 (0.09) | 34.41 (0.07) | 1.02 | | 41.85 (0.12) | 41.05 (0.12) | 40.26 (0.16) | 0.14 |  |
| Male, % | 47.74 | 55.48 | 61.63 | 0.28 | | 46.38 | 45.88 | 45.24 | 0.02 |  |
| Han ethnicity, % | 96.72 | 95.87 | 94.63 | 0.10 | | 96.14 | 96.01 | 95.78 | 0.02 |  |
| Married, % | 92.57 | 85.83 | 72.19 | 0.54 | | 85.60 | 84.49 | 84.26 | 0.04 |  |
| Education >12 y, % | 72.52 | 72.47 | 76.22 | 0.09 | | 56.15 | 56.42 | 56.86 | 0.01 |  |
| Alcohol use, % | 19.72 | 29.92 | 41.26 | 0.47 | | 29.52 | 31.21 | 31.97 | 0.05 |  |
| Smoking, % | 14.73 | 24.90 | 32.80 | 0.42 | | 25.42 | 26.88 | 27.57 | 0.05 |  |
| Adequate physical activities, % | 47.77 | 31.95 | 18.63 | 0.62 | | 34.54 | 32.75 | 29.38 | 0.11 |  |
| Employment-based medical insurance, % | 78.68 | 75.95 | 74.86 | 0.09 | | 58.57 | 58.12 | 58.01 | 0.01 |  |
| Diabetes, % | 2.89 | 2.21 | 0.92 | 0.14 | | 2.37 | 2.17 | 1.92 | 0.03 |  |
| Hypertension, % | 8.57 | 5.86 | 2.90 | 0.24 | | 6.34 | 5.81 | 5.67 | 0.03 |  |
| Hunan Center^₤^, % | 87.65 | 87.35 | 87.25 | 0.01 | | 87.39 | 87.50 | 86.85 | 0.02 |  |
| 24-month analytic sample (Cohort participants with BA measured at 22–26 months, n= 43,589) | | | | | | | | | | |
|  | N=15,074 | N=14,398 | N=14,117 |  | | Σw =13,757, rESS=56.6% | Σw =14,286, rESS=74.1% | Σw =14,140, rESS=43.3% |  |  |
| Age, years (mean (SE)) | 45.53 (0.10) | 40.85 (0.09) | 34.75 (0.08) | 1.01 | | 42.00 (0.13) | 41.21 (0.13) | 40.51 (0.18) | 0.13 |  |
| Male, % | 47.33 | 55.28 | 62.01 | 0.30 | | 53.68 | 53.90 | 54.48 | 0.02 |  |
| Han ethnicity, % | 96.83 | 96.43 | 95.37 | 0.08 | | 95.70 | 96.29 | 95.49 | 0.04 |  |
| Married, % | 93.51 | 87.44 | 74.22 | 0.53 | | 86.24 | 84.71 | 84.10 | 0.06 |  |
| Education >12 y, % | 74.34 | 74.32 | 79.34 | 0.12 | | 43.76 | 44.33 | 43.15 | 0.02 |  |
| Alcohol use, % | 19.62 | 29.94 | 41.19 | 0.47 | | 29.56 | 31.00 | 31.66 | 0.05 |  |
| Smoking, % | 15.46 | 25.22 | 33.99 | 0.43 | | 26.21 | 26.54 | 27.19 | 0.02 |  |
| Adequate physical activities, % | 47.35 | 31.91 | 18.30 | 0.62 | | 34.47 | 33.19 | 29.15 | 0.11 |  |
| Employment-based medical insurance, % | 80.30 | 78.21 | 77.97 | 0.06 | | 58.49 | 57.89 | 58.66 | 0.02 |  |
| Diabetes, % | 2.62 | 2.03 | 0.87 | 0.13 | | 2.42 | 2.15 | 2.07 | 0.02 |  |
| Hypertension, % | 8.01 | 5.92 | 2.81 | 0.23 | | 6.11 | 6.01 | 5.47 | 0.03 |  |
| Hunan Center^₤^, % | 87.84 | 87.24 | 87.31 | 0.02 | | 87.42 | 87.37 | 86.95 | 0.01 |  |

*Max pairwise SMD indicates the largest absolute SMD among the three dietary pattern groups. SMD < 0.10 indicates good balance (0.10–0.20 acceptable).

^₤^ Study center was not included in the IPTW model but SMDs were presented to describe its distribution across dietary pattern groups.

^╪^Weighted columns use the final stabilized analysis weights, calculated as the product of stabilized IPTW with year-specific IPCW weight with 1st–99th percentile truncation.

Abbreviations: IPTW, inverse probability of treatment weighting; IPCW, inverse probability of censoring weighting; SMD, standardized mean difference; Σw, sum of weights; rESS, relative effective sample size; SE, standard error.

| **Table S11. Associations between PCA derived dietary factors and ΔKDM-BA at 12- and 24-months** * | | | | |
| --- | --- | --- | --- | --- |
| Per-level increase in dietary factor scores^╪^ | ΔKDM-BA at 12 months | | ΔKDM-BA at 24 months | |
|  | β (years) (95%CI) | *P* value | β (years) (95%CI) | *P* value |
| Factor 1: irregular/discretionary eating & salty-eating-out factor | 0.043 (0.026, 0.061) | <0.001 | -0.013 (-0.069, 0.044) | 0.662 |
| Factor 2: low quality diet factor | 0.023 (0.009, 0.036) | <0.001 | -0.064 (-0.117, -0.010) | 0.020 |
| Factor 3: high-fat animal foods preference factor | 0.055 (0.041, 0.070) | <0.001 | -0.063 (-0.119, -0.008) | 0.025 |

*Weighted GEE model including both 12-month and 24-month time points with Gaussian identity link was used for association estimation and the analysis weights = year-specific IPCW only; Model was adjusted for baseline CA, gender, ethnicity, marital status, education, alcohol use, smoking, physical activities, medical insurance, diabetes, hypertension and study center. β>0 indicates higher BA at follow-up compared to baseline (years);

^╪^ Each factor score was divided according to the tertiles and coded 1-3; per-level increase reflects one tertile increment in factor score; Factor 1: Irregular/ discretionary eating & salty-eating-out factor; Factor 2: Low-quality diet; Factor 3: high-fat animal foods preference.

Abbreviation: PCA, Principal component analysis; KDM, Klemera and Doubal Method; BA, biological age; ΔKDM-BA: change in KDM-BA at follow up compared to baseline KDM-BA; CA, chronological age;

| **Table S12. Sensitive analysis on the associations of dietary pattern with change in biological age (ΔKDM-BA) at 12- and 24- months and interactions with time among population without hypertension or diabetes mellitus (n= 66,446)** | | | | | | | |
| --- | --- | --- | --- | --- | --- | --- | --- |
| Exposure | ΔKDM-BA at 12 months* | | | ΔKDM-BA at 24 months* | | | *P* for dietary pattern × time ^┼^ |
|  | β (years) | 95%CI | *P* value | β (years) | 95%CI | *P* value |  |
| Dietary pattern risk level as ordinal exposure^╪^ | | |  |  |  |  |  |
| Per-level increase in dietary pattern risk^╪^ | 0.019 | (0.005, 0.033) | 0.008 | 0.009 | (-0.052, 0.070) | 0.770 | 0.747 |
| Dietary pattern as categorical variable | |  |  |  |  |  |  |
| Low-risk dietary pattern | Reference |  |  | Reference |  |  | 0.758 |
| Moderate-risk dietary pattern | 0.045 | (0.019, 0.070) | < 0.001 | 0.002 | (-0.111, 0.115) | 0.967 |  |
| High-risk dietary pattern | 0.038 | (0.010, 0.066) | 0.007 | 0.018 | (-0.103, 0.139) | 0.771 |  |

* the sensitive analysis subgroup population was those without history of hypertension or diabetes mellitus, therefore, IPTW was remodeled with baseline CA, gender, ethnicity, marital status, education, alcohol use, smoking, physical activities and medical insurance; weighted GEE model including both 12-month and 24-month time points with Gaussian identity link was used for association estimation and the final analysis weights = stabilized IPTW × year-specific IPCW weight; model was adjusted for chronological age at baseline and study center. β>0 indicates higher BA at follow-up compared to baseline (years);

^╪^ Ordinal exposure treats dietary pattern as 0 =low-risk, 1=moderate-risk and 2=high-risk; Per-level increase indicates 0 increases to 1; or 1 to 2;

^┼^ *P* for interaction was estimated through the Type-III Wald test;

Abbreviation: KDM, Klemera and Doubal Method; BA, biological age; ΔKDM-BA: change in KDM-BA at follow up compared to baseline KDM-BA; CA, chronological age;
